# Using Caffeine Pills for Performance Enhancement. An Experimental Study on University Students’ Willingness and Their Intention to Try Neuroenhancements

**DOI:** 10.3389/fpsyg.2016.00101

**Published:** 2016-02-09

**Authors:** Ralf Brand, Helen Koch

**Affiliations:** Sport and Exercise Psychology, University of PotsdamPotsdam, Germany

**Keywords:** attitude, prevalence information, prototype-willingness-model, social reactivity, doping

## Abstract

Recent research has indicated that university students sometimes use caffeine pills for neuroenhancement (NE; non-medical use of psychoactive substances or technology to produce a subjective enhancement in psychological functioning and experience), especially during exam preparation. In our factorial survey experiment, we manipulated the evidence participants were given about the prevalence of NE amongst peers and measured the resulting effects on the psychological predictors included in the Prototype-Willingness Model of risk behavior. Two hundred and thirty-one university students were randomized to a high prevalence condition (read faked research results overstating usage of caffeine pills amongst peers by a factor of 5; 50%), low prevalence condition (half the estimated prevalence; 5%) or control condition (no information about peer prevalence). Structural equation modeling confirmed that our participants’ willingness and intention to use caffeine pills in the next exam period could be explained by their past use of neuroenhancers, attitude to NE and subjective norm about use of caffeine pills whilst image of the typical user was a much less important factor. Provision of inaccurate information about prevalence reduced the predictive power of attitude with respect to willingness by 40-45%. This may be because receiving information about peer prevalence which does not fit with their perception of the social norm causes people to question their attitude. Prevalence information might exert a deterrent effect on NE via the attitude-willingness association. We argue that research into NE and deterrence of associated risk behaviors should be informed by psychological theory.

## Introduction

Neuroenhancement (NE) is non-medical use of psychoactive substances or technology for the purpose of producing a subjective enhancement in psychological functioning and experience (Jongh et al., 2008; Mueller and Schumann, 2011; de Berker et al., 2013; Kipke, 2013; Clark and Parasuraman, 2014; Farah et al., 2014; Maier and Schaub, 2015, for elements of this definition). Sometimes the term ‘pharmacological cognitive enhancement’ is used to refer to the use of psychoactive drugs to enhance psychological capacities such as attention, concentration, and memory (e.g., Franke and Lieb, 2010; Dietz et al., 2013). The performance-enhancing effects of drugs used as neuroenhancers seem to be limited, regardless of whether they are prescription drugs such as methylphenidate (Ritalin), mixed amphetamine salts (Adderall) and modafinil (Provigil); illicit drugs (e.g., amphetamine, cocaine, ecstasy) or over-the-counter products (e.g., caffeinated drinks, energy drinks, *Ginkgo biloba*; e.g., Franke et al., 2014). Given the considerable overlap between the inconsistent cognitive effects of most of these substances on cognitive capacity and their incidental mood- and motivation-related effects (e.g., Ilieva et al., 2013) describing them as ‘cognitive enhancers’ may be overly narrow and restrictive (Zohny, 2015).

Wolff and Brand (2013) pointed out that information about the type and effects of a substance does not explain why people do or do not experiment with neuroenhancers. They argued that motivation to experiment with neuroenhancers could be explained by expectations about their effects. Someone drinking a strong cup of coffee explicitly in order to stay awake and thus study for longer or to enhance specific cognitive capacities (e.g., attention, recall memory) thus provides an adequate example of attempted NE. In this article, we will treat NE as goal-directed behavior intended to produce an improvement in academic performance.

There is an active debate about the ethics and pros and cons of NE at present and in the future (e.g., Hildt and Franke, 2013). Scientists remind us not to overestimate the effects of substances used as neuroenhancers (e.g., Franke et al., 2014; Zohny, 2015), but the market for substances (e.g., soft-drinks) promising an energy boost, or cognitive benefits as well as mood enhancement (Ishak et al., 2012) is increasing. In the light of these developments it is especially important to avoid fuelling the media hype surrounding NE (Partridge et al., 2011) and to state clearly the aims and limitations of published research. This paper is on the psychology of NE. We intend to describe and analyze university students’ motivation to experiment with NE in terms of a social cognitive theory of behavior.

### Prevalence of NE in Academia

There is limited reliable evidence on prevalence of NE in academia. Extant studies differ with regard to underlying definitions (e.g., whether NE is restricted to use of prescription drugs), representativeness of the sample (*ad hoc*; random), the types of substance considered (over-the-counter preparations; prescription drugs; illegal substances), data collection method (e.g., extent to which the confidentiality of self-reports is guaranteed) and time period investigated (e.g., point vs. life-time prevalence; Franke et al., 2014). Based on self-report questionnaires administered to large samples it has been reported that 5% in the year 2010/2011 (Middendorff et al., 2012) and 6% in 2014/2015 (Middendorff et al., 2015) of German university students abuse prescription drugs or illicit substances for “brain doping” during their years of study. Estimates of prevalence based on randomized response techniques that help to ensure the confidentiality of responses vary from 4% (Middendorff et al., 2015) to as much as 20% for 1-year prevalence in a study including caffeine pills as a neuroenhancer (Dietz et al., 2013). This latter figure is very similar to the reported abuse of prescription drugs for NE by research professionals (Maher, 2008). Maher reported that 20% of *Nature* readers from 60 nations who responded to an informal survey confessed to having used a prescription drug for NE at least once; however, Wiegel et al. (2015) reported that the lifetime prevalence of self-reported NE in a large sample of German university teachers and professors was only 1%.

This study focused on university students’ use of caffeine pills for NE. To date there has been only one pilot study of university students’ use of coffee, caffeinated drinks, and caffeine pills in Germany (Franke et al., 2011). The authors concluded that 10.5% of university students had used over-the-counter caffeine pills as neuroenhancers on at least one occasion.

### Psychological Factors Explaining NE in Students

Several studies have analyzed students’ motivation for NE. Eickenhorst et al. (2012) investigated German university students’ motives for using prescription drugs and illegal substances as neuroenhancers. The main motives reported by their participants were a desire to enhance concentration and alertness or to increase cognitive functioning in general; to enable them to relax, cope with stress and withstand performance pressure and a fear of being disadvantaged if they did not use such substances. Middendorff et al. (2012, 2015) reported that when interviewed most students indicated that their main motive for NE was to maintain, rather than enhance performance and that use of prescription drugs and over-the-counter products was particularly frequent during exam preparations.

Some authors have tried to go beyond descriptions of users’ motives and elucidate the psychological processes underlying NE behavior. Wolff and Brand (2013) regressed students’ NE behavior (use of prescription drugs and over-the-counter products) on their underlying positive attitude to NE and Sattler and Wiegel (2013) reported that six-month prevalence of prescription drug NE was higher in students with high test anxiety.

Three studies investigated the influence of selected psychological factors on participants’ decisions about trying NE using online factorial questionnaires (pseudo-experimental designs; see “Materials and Methods” section in this article). Sattler et al. (2013b) explored university students’ and teachers’ rationales for use of neuroenhancers using a hypothetical scenario in which they systematically varied several variables (indicated in italics): “A university [teacher/student] considers trying to enhance his cognitive performance for his [work/studies] by using a prescription drug which he does not require on medical grounds. He would be able to get the pills for free. A study that found that there is a *60 percent* chance that the drug will improve cognitive performance by *250 percent* caught his attention. The side effects were investigated: using the medication causes *slight* headaches in *one out of 100,000* users. Possible additional side effects are *unknown.*” Participants were asked to rate how willing they would be to use the substance described for NE if they were in the position of the person in the scenario. The authors controlled for several factors which they thought might influence decisions (e.g., moral evaluation of NE, conceptualized as the participant’s internal norm). The authors, e.g., found that the probability and severity of side-effects were negatively associated with willingness to use NE; internalization of social norms against use of neuroenhancers was also negatively associated with willingness to use NE. Sattler et al. (2013a) reported that NE was subject to a contagion effect: peer pressure (apparently high prevalence of substance use amongst peers) increased participants’ willingness to use NE whereas formal prohibition and provision of information about health side-effects decreased willingness. Sattler et al. (2014) investigated drivers of and obstacles to drug use in university students from the perspective of economic decision theory, assuming that decisions about NE would be based on a rational evaluation of the probability that a substance would help users attain their goals. Two thirds of their participants staunchly refused to use a prescription drug for NE, a similar proportion to that found in the two previous studies (Sattler et al., 2013a,b). Low intrinsic motivation, high test anxiety and previous use of drugs for NE were associated with greater willingness to use NE in the future. As in earlier studies willingness to use neuroenhancers was negatively associated with high internalization of social norms against NE (i.e., moral disapproval of NE; see above) and positively associated with apparent peer prevalence.

### Call for Theory-Driven Analysis of NE Behavior

Neuroenhancement is a multifaceted phenomenon (e.g., goals; factors governing decisions about use) that requires explanation at several levels (e.g., personal; social; environmental) based on a sound understanding of underlying psychosocial processes (e.g., evaluation of peer behavior). To date, however, most psychological research has been limited to identifying variables correlated with use of neuroenhancers and appears not to have been informed by psychological theory. Theory is an essential element of behavioral research; in particular the development of effective interventions is underpinned by explanatory models of behavior (Fishbein and Cappella, 2006). Experimental testing of theoretically derived hypotheses is superior to theoretically less-informed approaches, because it minimizes the risk of encountering arbitrary effects and overestimating the influence of variables (e.g., because of variable and case sampling errors).

This study aimed to address this research gap. It was based on the empirical findings from earlier psychological studies, but used an established theory of behavior to derive a model of the psychological determinants of NE behavior (cf. Zelli et al., 2015). Two other studies have used psychological theory to inform analyses of NE behavior. Wolff et al. (2014) used Job Demands Resources Theory to show that use of NE had a negative impact on university students’ psychological perceptions of academic demands and interfered with their intrinsic motivation and Wolff et al. (2013) used the strength model of self-control to predict first use of NE. This study tested predictions derived from the Prototype Willingness Model (PWM; Gibbons et al., 1998). We investigated how information about peer behavior influenced associations between the psychological variables predicting university students’ willingness and intention to use caffeine pills to enhance cognitive performance.

### The Prototype-Willingness Model

At the core of the PWM (Gibbons et al., 1998) is the idea that sometimes persons will find themselves in situations which facilitate but do not compel particular behaviors. The model postulates that under such circumstance behavior is determined by the individual’s willingness to perform the behavior in question rather than by a process of reflection with resulting plans and an intention at its end. Gibbons et al. (1998) defined willingness as an individual’s openness to risk opportunity, i.e., to perform a risky behavior in the absence of a specific plan or intention to do so. Although willingness may be accompanied by a congruent intention this is not necessarily the case. According to the PWM willingness is a foundation from which overt behavior can emerge spontaneously and it can, at least temporarily, sustain that behavior (Gerrard et al., 2008).

**FIGURE 1:**
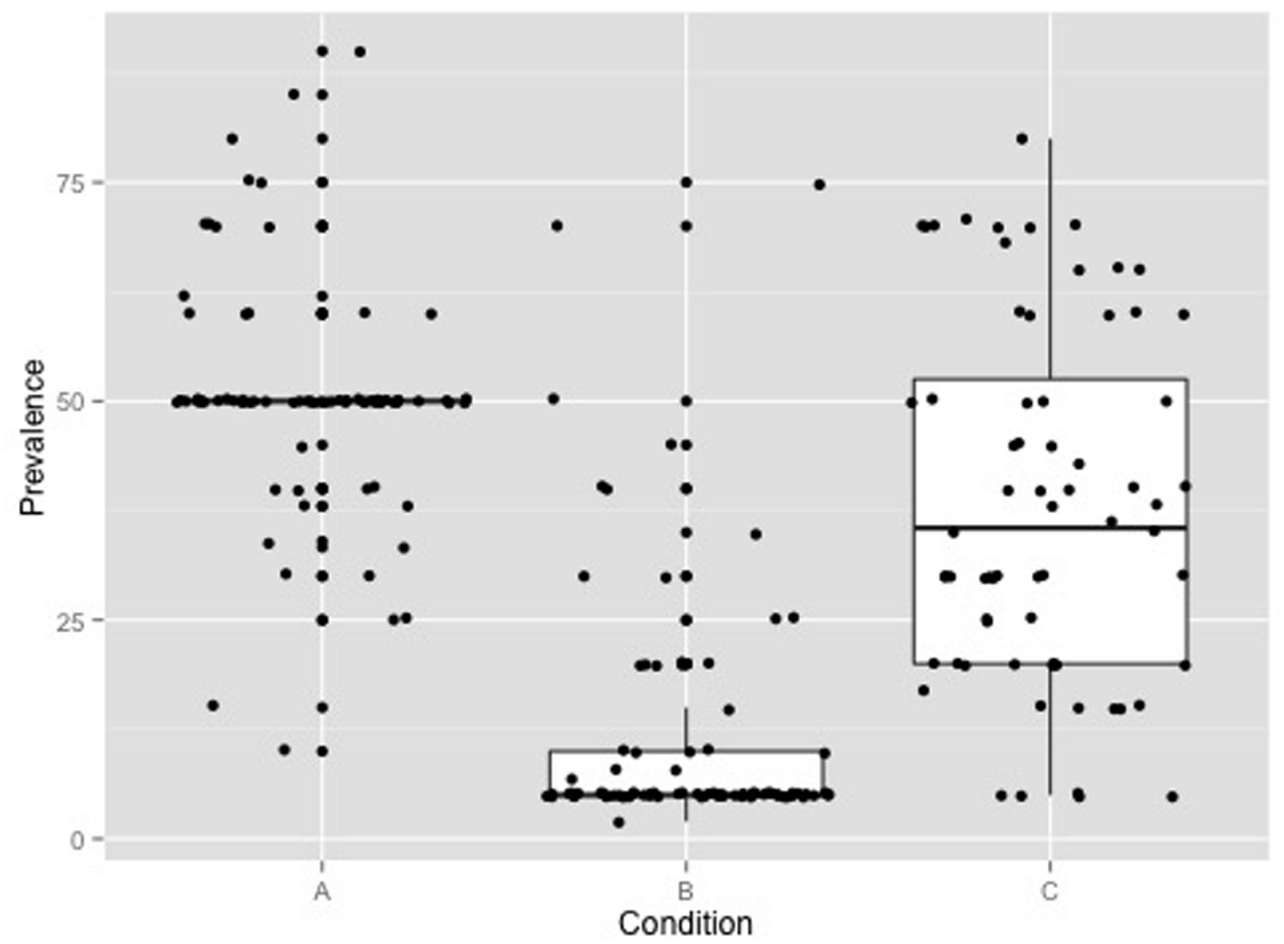
**Raw data and boxplots for participants’ answers to the question in manipulation check 1 (“According to recent research what percentage of university students in Germany have used caffeine pills at least once to enhance their performance and improve their learning?”) in experimental conditions A (50% peer prevalence), B (5%), and C (no information on peer prevalence)**.

The PWM is rooted in empirical analyses of adolescent risk-taking behavior and developmental health psychology theory. The model suggests two pathways for behavioral regulation, the reasoned pathway, which culminates in an intention and the social reactive pathway, from which a degree of willingness emerges. The two pathways are connected in various ways, most importantly through a postulated predictive relationship between willingness and intention (but not *vice versa*). The PWM is illustrated in full in **Figure 1**.

The PWM assumes that future behavior is informed by past behavior. The variable ‘past behavior’ is a factor in all processes or steps of both the reasoned and social reactive pathways, which respectively, influence ‘willingness’ and ‘intention’ to perform a given behavior (Gibbons et al., 2009).

All variables in the reasoned pathway are drawn from the Theory of Reasoned Action (Fishbein and Ajzen, 1975, 2010) which treats ‘attitude’ as the sum of evaluations of a psychological object captured by evaluations of attribute dimensions such as good-bad, harmful-beneficial, pleasant-unpleasant and likeable-dislikable (Ajzen, 2006). Another variable involved in the reasoned pathway is ‘subjective norm,’ which is defined as the perceived social pressure to engage or not engage in a given behavior. The PWM refers to descriptive norms (Gibbons et al., 1998). Items used to assess subjective norms should therefore capture individuals’ perceptions of what significant others do (e.g., “Do you think your peers would use caffeine pills to enhance cognitive performance?”) rather than what significant others think the respondent should do (injunctive norm; e.g., “Do you think that your peers would accept you taking caffeine pills for cognitive enhancement?”). ‘Intention,’ which is the PWM’s most proximal predictor of behavior of the reasoned pathway, is based on a combination of attitude and descriptive norm.

The social reactive pathway is defined by certain variables’ relationships with willingness. ‘User prototype’ is one of these variables. Gerrard et al. (2008) recommended that this variable should be measured using items such as “Take a moment to think about what kind of student in your age group uses caffeine pills to enhance cognitive functioning. We are not interested in anyone in particular, just the typical person of your age who could do this. How [popular/smart/selfish] is this person?” In the context of the PWM a ‘user prototype’ is thus the sum of the characteristics of an imagined peer who engages in the target behavior (e.g., the ‘typical’ smoker). The social acceptability of a user prototype is positively associated with willingness to engage in the target behavior. ‘Subjective norms’ influence behavior via the reasoned pathway (through their association with ‘intentions’) and via the social reactive pathway, where they are postulated to be associated with ‘willingness.’ The socially reactive nature of the willingness construct is underscored by Gerrard et al.’s (2008) recommended items for measuring it, e.g., “Suppose you were studying with a group of friends and there were caffeine pills available. How willing would you be to take one?”

In the past few years, research on the PWM has accumulated in health psychology and a meta-analysis was published very recently (Todd et al., 2014). One of the conclusions from this meta-analysis was that the predictive power of the relationships postulated by the PWM is only marginally better in adolescent samples (*R*^2^ = 0.33) than in adult samples (*R*^2^ = 0.29), suggesting that although the model was developed to predict risk-taking behavior in adolescents it may be just as useful for predicting adult behavior. It is important to note that the meta-analysis did not test the full set of relationships involving PWM variables; it focused on the postulated correlations between prototypes, willingness and intentions and thus corroborated the postulated processes of the social reactive path. Most importantly the meta-analysis revealed that although willingness and intention are sometimes highly correlated they also act as independent predictors of risk behavior. Willingness explained an additional 4.9% of variance in behavior after intentions had been taken into account (Todd et al., 2014). The postulated correlations between variables in the reasoned pathway have been tested in several other PWM-related studies (e.g., the association between past behavior and attitude, Gibbons et al., 1998; pairwise associations between norms and intentions and willingness, Gerrard et al., 2008) and in meta-analyses which have confirmed the pattern of relationships among attitude, norms, intentions, and behavior (Armitage and Conner, 2001) postulated in the Theory of Planned Behavior (Ajzen, 1991).

Astonishingly, however, there is a dearth of empirical analyses of the full set of PWM variables using, e.g., multivariate model testing methodology.

### This Study

Neuroenhancement research (e.g., Wolff and Brand, 2013; Schelle et al., 2014), research in related domains (e.g., doping in sport, Ntoumanis et al., 2014) and behavior change research more generally (e.g., McEachan et al., 2011) have shown that variables used in social cognitive theories, especially attitude, perceived social norms, and intentions (e.g., Fishbein and Ajzen, 1975, 2010), are important psychological predictors of behavior. Recently university students’ willingness to use psychoactive substances for performance enhancement emerged as an empirically useful dependent variable in NE research (e.g., Sattler et al., 2013b). The PWM is a comprehensive psychological theory of behavior that integrates all these variables. The PWM postulates two interacting pathways of behavioral regulation: the reasoned action path, in which attitudes and norms predict intention (the most proximal predictor of behavior) and the social reactive path, which integrates information from prototypes, attitudes, and norms to form another proximal predictor of behavior, i.e., behavioral willingness. The PWM thus represents an integrative psychological framework for the study of NE behavior.

Earlier research indicated that 10% of German university students use caffeine-containing products with the purpose to enhance cognitive performance on at least one occasion during their student life (Franke et al., 2011). Use of drugs for NE carries health risks (Schermer et al., 2009) and students consider it a risky behavior (Sattler et al., 2014). Peer behavior and peer pressure have been shown to be factors in university students’ decisions about use of enhancers (Sattler et al., 2013a).

In our investigation, we sought to manipulate groups of university students into believing either that use of caffeine pills for NE was rather widespread amongst their peer group (experimental condition A: prevalence given as 50%; i.e., five times more than the actual estimated prevalence) or rare (condition B: prevalence given as 5%; i.e., half the estimated prevalence). A third group received no information about prevalence and served as a control group (condition C). We investigated use of caffeine pills specifically. Caffeine is available in over-the-counter products and in higher doses as a prescription drug. We deliberately restricted our investigation to use of caffeine in pill form, because we assumed that such products were more likely to be seen as ‘pharmaceuticals’ than, for example, caffeinated energy drinks which might be perceived as an everyday consumable.

First of all, we tested whether the PWM could be used to describe university students’ willingness and intentions with respect to use of NE. Our second research question was based on the assumption that university students would have some sort of subjective perception of the prevalence of NE in their peer group and that this perception would shape their thinking about NE. We explored whether experimentally manipulating information about the prevalence of use of caffeine pills for NE amongst peers (high prevalence; low prevalence; no prevalence information) would influence the behavioral determinants specified in the PWM (attitude, subjective norm, user prototype, willingness, and intention) and the associations between these variables and willingness or intention to engage in the target behavior.

## Materials and Methods

This study was conducted online as a factorial survey experiment (Jasso, 2006). This method enables the researcher to determine the influence of experimentally manipulated information on respondents’ self-reported thoughts, feelings, and decisions. The Questback EFS 10.6 software was used to run the experiment. The questionnaire was presented in German (i.e., all example items below are English translations from the German originals). The access link was distributed via Facebook and email thus creating a convenience sample drawn from the target population, university students in Germany. Data collection started in March 2015 and ended 3 weeks later in April 2015. The study was carried out in accordance with the recommendations of the ethical committee of the University of Potsdam (February 2015). All participants gave written, informed consent in accordance with the Declaration of Helsinki.

### Participants

Four hundred and thirty-six university students clicked on the link giving access to our survey. Of these 386 (88.5%) went beyond the title page and 305 (69.9%) completed the questionnaire. Forty-nine students did not consent to having their data saved and analyzed on the last page, so the responses of 256 participants (58.7% of those who clicked on the link) were stored for analysis. Twenty-five participants had provided incomplete data (>40% missing responses) and were therefore excluded from the final analyses, so the final sample consisted of data from 231 German-speaking university students of whom 75.8% were women (*M* age = 23.5 years ± 2.7; range: 18–35 years) and 23.8% were men (*M* age = 25.4 years ± 3.8; range: 20–40 years). One 24-year-old participant did not provide information about gender.

### Experimental Manipulation

When they clicked through to the second page of the online questionnaire participants were randomly assigned to one of the three experimental conditions. Participants in condition A (fivefold overestimation of prevalence; Franke et al., 2011) read the following, “A few months ago a representative study showed that more than 50% of all university students in Germany use caffeine pills in order to enhance cognitive functioning and improve learning.” Participants in condition B (i.e., 50% underestimation of prevalence) read that only 5% of students used caffeine pills for NE. Participants in condition C read, “A few months ago a representative study showed that some university students in Germany use caffeine pills to enhance cognitive functioning and improve learning.”

### Measures and Information

#### Past Behavior

This indicator was intended to capture participants’ historical NE behavior. They were asked four yes-no questions: “Have you ever used caffeine pills to enhance your cognitive performance and improve your learning?,” “Have you ever used a caffeinated synthetic drink (‘energy drink’) to enhance your cognitive performance and improve your learning?,” “Have you ever used any other synthetic substance to enhance your cognitive performance and improve your learning?,” and “Have you ever drunk a cup of strong coffee or tea to enhance your cognitive performance and improve your learning?” Responses to these four questions were analyzed separately to characterize the sample, but the mean score was used in the main statistical analyses. McDonald’s ω_h_ = 0.52 that takes into account the dichotomous nature of these heterogeneous set of items indicated adequate general factor saturation.

#### Subjective Norm

The descriptive facet of this PWM variable was captured using three statements (see Hammer and Vogel, 2013) to which participants responded using a six-point, i.e., forced choice Likert-type scale (1 = “totally disagree” to 6 = “totally agree”): “Peers whose opinions I value use caffeine pills to enhance their cognitive performance and improve their learning,” “People who are important to me would use caffeine pills to enhance their cognitive performance and learning if they were in my position,” and “People around me have used caffeine pills to enhance their cognitive performance and improve their learning.” The mean score for all three statements was used in the statistical analyses. In our sample the internal consistency of this scale was α = 0.81.

#### Attitude

Participants used a seven-point scale to rate use of caffeine pills to enhance cognitive performance and improve learning on five semantic differentials: ‘bad–good,’ ‘unhealthy–healthy,’ ‘right–wrong,’ ‘risky–safe,’ and ‘useless–useful’ (see Ajzen, 2006). Higher values represented a more positive attitude toward use of caffeine pills for NE. The mean score from these five items was used for statistical analyses. In our sample the internal consistency of the scale was α = 0.80.

#### User Prototype

Participants were asked to imagine what a typical user of caffeine pills – perhaps a fellow student from their university – might be like (see Gibbons et al., 1998) and use a seven-point scale to describe that person in terms of five semantic differentials ‘stupid–smart,’ ‘unpopular–well-liked,’ ‘motivated–unmotivated,’ ‘effective–ineffective,’ and ‘wrong–right.’ The mean score for the scale was used in statistical analyses; higher means indicated greater social acceptability. In our sample the internal consistency of the scale was α = 0.75.

#### Willingness

Participants were asked to read two hypothetical scenarios and rate their willingness to use NE in each (see Gibbons et al., 1998). In scenario one, they read: “Suppose that a friend from your study program has written on Facebook that he recently used caffeine pills from the drugstore to improve his learning.” Participants then rated how likely it was that they would take caffeine pills at some point using a six-point, i.e., forced choice Likert-type scale (1 = “very unlikely” to 6 = “very likely”). In scenario two, they read: “Usually you are well prepared for exams, but this time you feel tired and worn out. Although you are trying hard, you just don’t seem able to prepare well for the forthcoming exam. You have the option of taking caffeine pills to dispel your fatigue and thus revise more effectively.” Participants then responded to two questions (“If you had the opportunity, would you be willing to use caffeine pills?” and “Would you be willing to experiment with caffeine pills under some other circumstance if you had the opportunity to do so?”) using a six-point, i.e., forced choice Likert-type scale (1 = “absolutely not willing” to 6 = “perfectly willing.” A mean score was calculated and used for statistical analyses, with higher scores indicating greater willingness to use caffeine. In our sample the internal consistency of this scale was α = 0.88.

#### Intention

This variable was measured very simply, by asking participants the single specific question “Do you intend to use caffeine pills for cognitive enhancement and to improve learning in preparation for your next exams?” Responses were given on a six-point, i.e., forced choice Likert-type scale (1 = “definitely not” to 6 = “yes, definitely”).

#### Personal Details

Participants were asked to provide their age and gender, and state whether they were currently enrolled as a university student.

#### Manipulation Checks

After responding to all the scales described above and providing personal data participants were asked “According to recent research what percentage of university students in Germany have used caffeine pills at least once to enhance their performance and improve their learning?” The response was entered in a free-text input field. Participants were also asked whether they thought there was anything suspicious, wrong, or strange about the questionnaire, and in particular if they thought they had been manipulated by the ways in which we gave or asked for information earlier in the questionnaire.

#### Debriefing

The last page of the questionnaire provided full information about the goals and procedure for the study. This included a statement of the actual estimated prevalence of use of caffeine pills for NE among university students (i.e., 10%) and an explicit admission that we had tried to deceive our participants about this statistic in two experimental conditions. Participants had to tick response boxes to indicate that they had read and understood this information and consented to the confidential storage and analysis of their data for scientific use by the Division of Sport and Exercise Psychology of the University of Potsdam.

### Statistical Analyses

SPSS 22.0 was used to calculate all descriptive statistics and for tests for group differences (ANOVA, MANOVA) and frequency distribution tests (χ^2^ test). Structural equation modeling (SEM) with Amos 22.0 was used to determine whether our data were consistent with the PWM. General model fit was evaluated according to established criteria (*RMSEA* < 0.08, *CFI* ≥ 0.95, *SRMR* < 0.08; e.g., Hu and Bentler, 1999; Beauducel and Wittmann, 2005) and a Bollen–Stine bootstrap (1000 iterations) was used to estimate confidence intervals for regression weights. A SEM multigroup moderation approach (Byrne, 2010) was used to investigate experimentally induced alterations in the relative predictive power of PWM predictors of willingness and intention (Lowry and Gaskin, 2014). These tests were carried out with a program created by Gaskin (2012) which calculates *z*-scores based on critical ratio tests of the multigroup model and unstandardized estimates.

The significance level was set at *p* < 0.05 for all analyses. Information from the second question of the manipulation check was evaluated qualitatively.

## Results

### Randomization Checks

One-way ANOVA revealed no significant age differences between the three groups [*F*(2) < 1], indicating that in this respect the randomization procedure was successful. However, assessment of the gender ratios in the three groups indicated that there were fewer men (*n* = 12) and more women (*n* = 73) than expected in experimental condition B, χ^2^(2) = 7.82, *p* < 0.05. Previous research suggests that male and female students may differ in their use of caffeine for NE (Franke et al., 2011), so this randomization error might have resulted in group differences in ‘past behavior’; however, in practice there were no significant differences between conditions with regard to either historical use of specific substances (all *p*s *n.s.*; see below) or mean scores used in the statistical analyses (MANOVA results; see below). We therefore concluded that in spite of the randomization error (under-representation of men in condition B) the randomization process was successful overall.

### Manipulation Check

The group mean responses to the question about what percentage of university students in Germany used caffeine pills for NE on at least one occasion were as follows: group A (fivefold overestimate of prevalence, i.e., 50%) *M* = 50.39% (*SD* = 13.24), group B (50% underestimate of prevalence, i.e., 5%) *M* = 11.28% (*SD* = 13.88) and group C (no information about prevalence) *M* = 38.18% (*SD* = 20.34). This finding is illustrated in **Figure 1**. There were group differences in responses [univariate ANOVA, *F*(2,228) = 130.2, *p* < 0.01]. The participants’ free responses to the second manipulation check question indicated that participants did not see through the experimental manipulation.

### Description of Past Behavior

Thirty-one participants (13.4%) reported having used caffeine pills to enhance their performance on at least one occasion. High-dose synthetic caffeinated drinks (’energy drinks’) had been used by 39.0% (*n* = 90) and 14.3% (*n* = 33) reported having used some other synthetic substance for NE, whilst 77.1% (*n* = 178) had used a cup of strong coffee or tea, or some other natural substance for NE.

### Main Analyses

#### PWM Model Fit

Descriptive statistics for all variables are summarized in **Table 1**. The value of Mardia’s multivariate kurtosis statistic was 1.24, indicating the multivariate normality of our data. The low variance inflation factor of 1.7 suggested that multicollinearity could be dismissed as a possible source of bias.

**Table 1:** Descriptive statistics for all variables included in SEM.

Variables	*M*	(*SD*)	Range	Skewness	Kurtosis
Past behavior	1.36 (0.26)	1–2	0.68	0.12
Attitude	2.88 (1.04)	1–5.80	0.06	-0.23
Subjective norm	2.25 (1.19)	1–6	0.79	-0.47
User prototype	4.34 (0.90)	1–7	-0.09	0.40
Intention	2.13 (1.38)	1–6	1.04	0.08
Willingness	3.00 (1.39)	1–6	0.21	-1.08

*N = 231 for all variables*.

**FIGURE 2:**
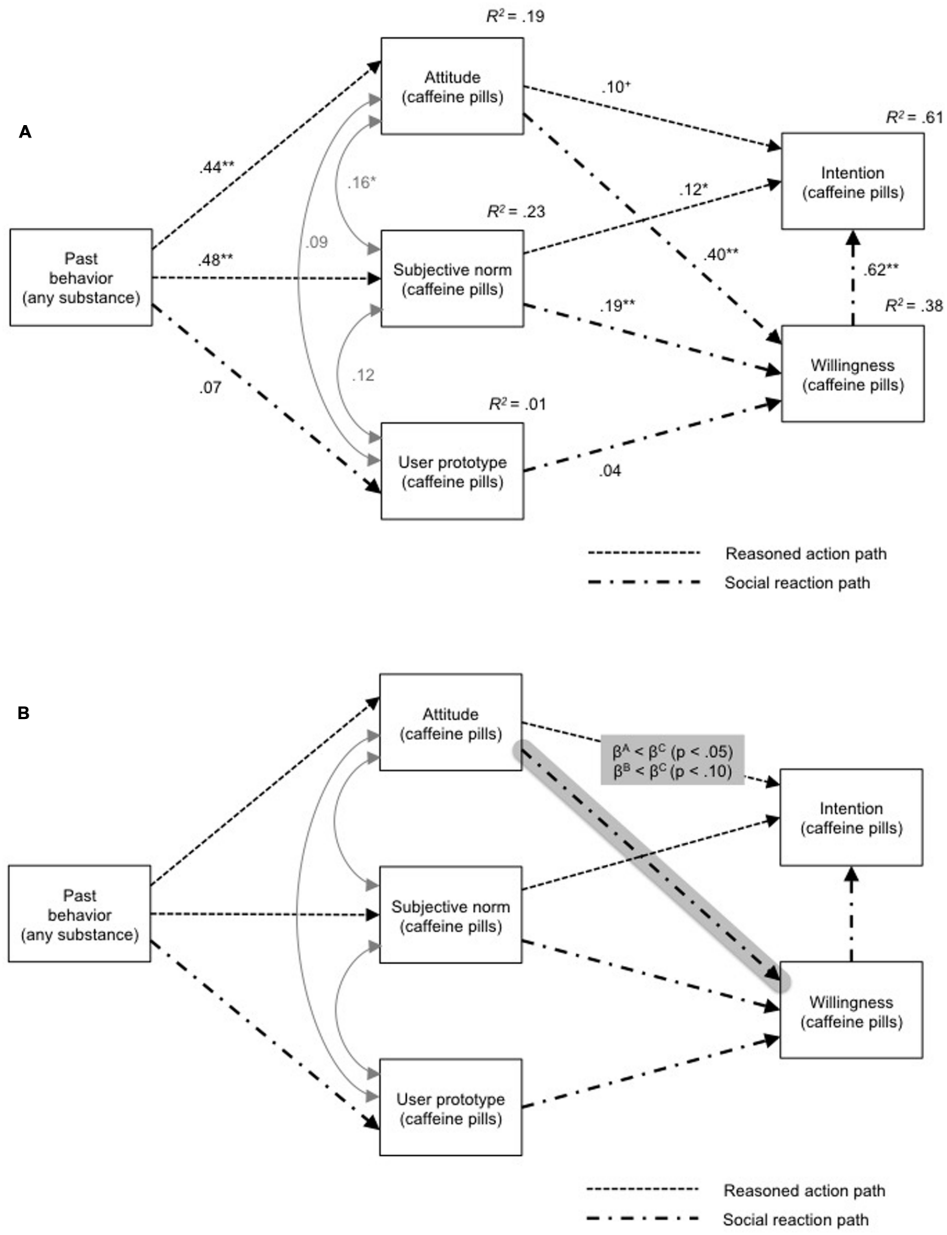
**The Prototype Willingness Model (Gibbons et al., 2009) with results from SEM in (A) (*R*^2^ for PWM variables and β_stand._ for paths) and the illustration of experimental effects (B)**.

Structural equation modeling indicated that our data were a good fit with the predictions of the PWM after we had taken into account additional correlations between ‘past behavior’ and the residual variance in ‘intention’ (*r* = 0.18, *p* < 0.05) and between ‘past behavior’ and the residual variance in ‘willingness’ (*r* = 0.30, *p* < 0.01), χ^2^(1) = 1.95, *p* = 0.16; *RMSEA* = 0.06; *CFI* = 0.99, *SRMR* = 0.01. The structural model with regression weights for relationships between variables (bootstrapped *CI*s in **Table 2**) and determination coefficients for variables is displayed in **Figure 2A**.

**Table 2:** Bootstrapped 95% *CI*s for regression weight (β_stand._).

Parameters			Lower boundary	Upper boundary
Past behavior	→	Subjective norm	0.37	0.58
Past behavior	→	User prototype	-0.08	0.19
Past behavior	→	Attitude	0.34	0.53
Subjective norm	→	Willingness	0.08	0.30
Attitude	→	Willingness	0.28	0.50
User prototype	→	Willingness	-0.06	0.14
Subjective norm	→	Intention	0.01	0.24
Attitude	→	Intention	-0.01	0.21
Willingness	→	Intention	0.51	0.72

*N = 231 for all variables*.

Regression weights for the association between ‘attitude’ and ‘intention’ in the reasoned action pathway and the sequence of associations linking ‘past behavior’ to ‘user prototype’ to ‘willingness’ in the social reaction path were all low (all β_stand._ ≤ 0.10). ‘Willingness’ had a substantial effect on ‘intention’ (β_stand._ = 0.62). The model explained 38% of variance in ‘willingness’ and 61% of variance in ‘intention.’

In summary, our main hypothesis, that the PWM can be applied to university students’ willingness and intentions with respect to use of caffeine pills for NE, was supported.

#### Experimental Effects on Variable Means

The MANOVA omnibus test revealed no significant effect of experimental condition on the six PWM variables, *F*(12,448) = 0.91, *n.s.* We therefore concluded that provision of experimentally manipulated information about peer behavior did not produce group mean differences in any of the investigated variables.

#### Experimental Effects on Regression Paths

Model fit indices remained acceptable for the multigroup estimation model, χ^2^(3) = 6.85, *p* = 0.08; *RMSEA* = 0.07; *CFI* = 0.99, *SRMR* = 0.01. Provision of experimentally manipulated evidence about prevalence lowered regression weight for the associations between ‘attitude’ and ‘willingness’ in condition A by 45% relative to the control group, which did not receive any prevalence information; a similar effect (lowered regression weight by 40%) appeared as a statistical trend in condition B (**Figure 2B**; condition A: β_stand._ = 0.31, *p* < 0.01; condition B: β_stand._ = 0.34, *p* < 0.01; condition C: β_stand._ = 0.57, *p* < 0.01). This means that in our sample provision of information about prevalence of use of caffeine pills for NE decreased the predictive value of attitude to use of caffeine for NE on willingness to do so.

## Discussion

Testing predictions derived from a theory of behavior minimizes the risk of reporting random effects and overestimating relationships; it is thus a suitable strategy for uncovering the psychological mechanisms underlying behavior and behavior change (Zelli et al., 2015). This study showed that the PWM, an established social cognitive theory of behavior, can be used to describe university students’ willingness and intention to use caffeine pills to enhance their academic performance (NE). On the basis of this result and previous findings on the effects of peer pressure (Sattler et al., 2013a,b, 2014) we explored whether providing information about the prevalence of a behavior amongst peers – in this case use of caffeine pills for NE – would influence motivational variables and their interrelationships. We found that attitude was a less powerful predictor of willingness to use caffeine pills in experimental condition A, in which participants were informed that recent research indicated that 50% of all university students in Germany used caffeine for NE. There was a trend in the same direction (*p* < 0.10) in condition B, where participants were informed that only 5% of peers used caffeine for NE.

Interestingly, in this investigation attitude was a much weaker predictor of intention than in other investigations of problematic consumption behavior (e.g., *r*_+_ = 0.62 in a meta-analysis of intention to consume alcohol, Cooke et al., 2014; *r* = 0.55 in a meta-analysis of athletes’ intention to use doping substances, Ntoumanis et al., 2014). The most likely explanation for this is that we chose to measure a very specific intention, i.e., intention to use caffeine, in pill form, in the next exam period. The relatively skewed distribution (*z* = 1.04) of rather low intentions (*M* = 2.13 ± 1.38 on a six-point Likert scale) corroborates this explanation. Our participants’ willingness to use caffeine for NE was somewhat higher (*M* = 3.00 ± 1.39) and barely skewed (*z* = 0.21). We consider that our very straightforward way of measuring a very specific intention represents a valid, reliable indication of intention with respect to the behavior in question, we therefore argue that the ‘willingness’ construct is the more interesting proximal psychological predictor of NE (Sattler et al., 2014) and should be investigated further. In particular, we recommend research into the conceptual relationship between willingness and intention (empirical data from many studies based on the PWM suggest that the two constructs are fairly highly correlated, c.f. Todd et al., 2014; β_stand._ = 0.62 in our sample). We think that willingness to engage in a given behavior may be much more sensitive to changes in motivational predictors (e.g., in attitude) and to situational factors than an intention to do so. A fundamental assumption of the PWM is that willingness is the basis of socially reactive, i.e., ‘spontaneous’ or unplanned behavior; this suggests that willingness would be a suitable target for public health interventions.

Structural equation modeling modeling enabled us to estimate correlations between the PWM variable past behavior and the residual variance in willingness and intention with respect to the relevant behavior. In accordance with Wolff and Brand’s (2013) behavioral approach to investigating motivation to use NE we measured our participants’ past NE behavior without regard for the substances involved although we measured willingness and intention in relation to a specific substance, namely caffeine pills. Our data suggests that an individual’s general disposition to use neuroenhancers is a product of unobserved variables in addition to the PWM variables we measured. These unidentified variables have a considerable impact on willingness and intention to use specific neuroenhancers and perhaps NE more generally. Candidates for these thus far unidentified variables might be found in the psychological roots of NE behavior and perhaps in the goals at which NE is directed. More generally we endorse Lazuras’s (2015) recommendation, made in relation to research on doping in sport (see Zelli et al., 2015, for parallels between doping and NE), that researchers should develop coherent explanatory models that account for environmental influences, demographic variables, culture, and exposure to information in the media.

The PWM variable past behavior was linked to social norm and attitude but had almost no relationship with user prototype. Participants’ images of the “typical user” (Gibbons et al., 2009) of caffeine pills, including social acceptability (i.e., user prototype) also had little impact on willingness to use NE. In the terminology of the PWM the basis for socially reactive use of caffeine pills to improve academic performance seems to be the strong influence of attitude on willingness; user prototype appears to play little role in socially reactive NE in this instance. This may be due to our choice of example neuroenhancer, namely caffeine pills. Caffeine pills may be more likely than other caffeine products such as ‘energy drinks’ to be treated as a pharmaceutical that should not be taken recklessly, or in response to peer pressure. If we assume that university students are better educated than the general population we might expect them to be more resistant to social influences and generally less prone to unplanned, socially reactive behavior and hence that user prototype would be a stronger predictor of NE behavior in other samples and in relation to other substances.

The experimental manipulation of information about prevalence of use of caffeine for NE amongst peers (rather widespread; rare) did not affect participants’ subjective norm for this behavior, in other words quantitative information about prevalence did not appear to be internalized and incorporated into belief systems immediately. This may be because the information given in conditions A (50% prevalence, i.e., a fivefold overstatement of actual estimated prevalence) and B (5%, i.e., half the estimated prevalence) did not correspond with our participants’ existing perceptions based on personal observations. The responses of the university students in condition C (no information on peer prevalence) to the manipulation check question about prevalence (see **Figure 1**) provide some indication of students’ pre-existing perceptions of NE prevalence and suggest that such perceptions vary widely. We suggest that receiving new information about peer prevalence might weaken the association between attitude to a given behavior and willingness to engage in it. Our finding that in condition A the predictive power of attitude with respect to willingness decreased by 45% might be taken as an indication that our participants had begun to reflect on their point of view in response to the rather surprising – i.e., inaccurate, experimentally manipulated – information about the prevalence of NE amongst their peer group. This finding reinforces our main contention, shared by other authors (Wolff and Brand, 2013; Wolff et al., 2014; Zelli et al., 2015), which is that social cognitive theories which define relationships between, e.g., attitude and other psychological predictors of behavior (in this case NE) provide valuable insight into the psychological mechanisms underlying behavior change and hence can be used to develop behavior prevention programs.

The limitations of this research should be acknowledged. First of all, psychological theories such as the PWM are intended to predict behavior. We have neither predicted a temporal relationship (e.g., that past behavior influences attitude) nor measured observed behavior (e.g., use of caffeine pills in the next exam period) following an experimental treatment. We experimentally manipulated one variable (information about prevalence) and were thus able to make causal inferences related to this manipulation (providing information about prevalence reduced the influence of attitude to NE on willingness to engage in it). We are, however, unable to draw conclusions about the validity of theoretical assumptions about the causal relationships between other variables (e.g., the direction of the association between subjective norms and willingness; **Figure 1**) as our evidence on this was correlational. Longitudinal studies are needed to draw conclusions about the consequences of changes in motivational determinants. Another limitation of our study is that our analyses were based on data from an *ad hoc* sample of university students which may not have been representative of the population. Although, we are optimistic that our findings are valid further studies are needed to corroborate our findings and interpretation.

## Conclusion

We hope that future research will be theoretically informed, seeking to address research questions derived from and relevant to psychological theory. By taking this kind of approach we have shown that information about the prevalence of a behavior amongst peers – in this case use of NE to improve academic performance – might have a deterrent effect via attitude to NE and willingness to engage in NE. The approach described in this study might be particularly useful for the designers of public health campaigns.

## Author Contributions

RB developed this research question. HK conducted the empirical part of the study as a part of her bachelor thesis. RB and HK jointly re-analyzed the data, adjusted and broadened the chain of arguments, and then cooperatively wrote this report.

## Conflict of Interest Statement

The authors declare that the research was conducted in the absence of any commercial or financial relationships that could be construed as a potential conflict of interest. The reviewer Christopher Fullerton and handling Editor declared their shared affiliation, and the handling Editor states that the process nevertheless met the standards of a fair and objective review.
